# Virtual reality: a powerful technology to provide novel insight into treatment mechanisms of addiction

**DOI:** 10.1038/s41398-021-01739-3

**Published:** 2021-12-06

**Authors:** Massimiliano Mazza, Kornelius Kammler-Sücker, Tagrid Leménager, Falk Kiefer, Bernd Lenz

**Affiliations:** 1 Department of Addictive Behavior and Addiction Medicine, Central Institute of Mental Health (CIMH), Medical Faculty Mannheim, Heidelberg University, Mannheim, Germany; 2 Center for Innovative Psychiatric and Psychotherapeutic Research, Central Institute of Mental Health (CIMH), Medical Faculty Mannheim, Heidelberg University, Mannheim, Germany; 3 Department of Cognitive and Clinical Neuroscience, Central Institute of Mental Health (CIMH), Medical Faculty Mannheim, Heidelberg University, Mannheim, Germany

**Keywords:** Scientific community, Pathogenesis

## Abstract

Due to its high ecological validity, virtual reality (VR) technology has emerged as a powerful tool for mental health research. Despite the wide use of VR simulations in research on mental illnesses, the study of addictive processes through the use of VR environments is still at its dawn. In a systematic literature search, we identified 38 reports of research projects using highly immersive head-mounted displays, goggles, or CAVE technologies to provide insight into treatment mechanisms of addictive behaviors. So far, VR research has mainly addressed the roles of craving, psychophysiology, affective states, cognition, and brain activity in addiction. The computer-generated VR environments offer very realistic, dynamic, interactive, and complex real-life simulations requesting active participation. They create a high sense of immersion in users by combining stereoscopic three-dimensional visual, auditory, olfactory, and tactile perceptions, tracking systems responding to user movements, and social interactions. VR is an emerging tool to study how proximal multi-sensorial cues, contextual environmental cues, as well as their interaction (complex cues) modulate addictive behaviors. VR allows for experimental designs under highly standardized, strictly controlled, predictable, and repeatable conditions. Moreover, VR simulations can be personalized. They are currently refined for psychotherapeutic interventions. Embodiment, eye-tracking, and neurobiological factors represent novel future directions. The progress of VR applications has bred auspicious ways to advance the understanding of treatment mechanisms underlying addictions, which researchers have only recently begun to exploit. VR methods promise to yield significant achievements to the addiction field. These are necessary to develop more efficacious and efficient preventive and therapeutic strategies.

## Introduction

Addictive disorders are highly prevalent and very complex conditions. They pose strong burdens on the affected individuals, their relatives, and the society in general. Many patients with addictions show a chronic relapsing course and cannot maintain long-term abstinence. So far, the mechanisms underlying addictions are poorly understood. Also, we lack reliable, sufficiently accurate, and objective biomarkers of addictive behavior. To diagnose addictive disorders, clinicians mainly rely on self-report measures, which are subject to diverse biases. A better understanding of the interaction between biopsychosocial and environmental factors in the development and maintenance of addictive disorders is needed to improve the therapeutic outcome of substance use disorders (SUD).

Cognitive, behavioral, and physiological reactivity to environmental cues is a well-established phenomenon in the pathophysiology of addictions [1, 2]. Craving is an important cue-elicited behavior, as it provokes drug-seeking behavior with preparatory physiological responses [3] and ultimately relapses in so far successful abstainers [4]. Besides being a central characteristic of addictions, craving is also an important diagnostic criterion, and a major factor for the chronic relapsing course of addictive disorders [4–6]. It includes psychological, cognitive, emotional, physiological, and behavioral aspects related to desiring or “wanting” a drug or an experience with subsequent seeking behavior [7].

Craving is elicited by a vast myriad of triggers including the location and situation (i.e., at home, bar, restaurant, party), social cues, day time, weekday, emotional state, gender, and age [8]. Relapses are usually preceded by complex and inter-individually varying high-risk situations with powerful but often unknown cues. These involve visual, auditory, olfactory, and tactile perceptions, as well as social interactions. Many cue reactivity studies have investigated the effects of direct cues and neglected contextual triggers, as these are hard to simulate in laboratory and clinical settings. Thus, novel methods are necessary to gain deeper insight into how environmental factors trigger the individual’s addictive behaviors.

The available therapeutic strategies to empower patients with addictions to deal with craving, physiological responses, and dysfunctional cognitions with the goal to prevent relapse have limited efficacy. The same is true for cue exposure therapy, which is used to extinguish the strongly linked stimulus-response association of an addictive behavior and which can be combined with biofeedback training [9, 10]. The traditional in vivo methods using pictures and photographs, passive videos, olfactory scents, tactile materials such as wine glasses, bongs, and pipes and guided imagery programs offer only minor ecological validity. This explains the limited efficacy of today’s clinical settings; e.g., lab bars have been shown to induce lower expectancies of alcohol effects than real bars [11]. Moreover, patients may not be able to make use of behavioral strategies learned in cue-free clinical settings when they are in their natural cue-laden environments.

Mainly due to their high ecological validity, virtual reality (VR) technologies have emerged as very powerful tools to overcome the limitations of classical laboratory and clinical settings. In VR environments, individuals immerse in a computer-generated world that reacts to the individual motions and behaviors. In comparison to in vivo settings, VR enhances the possible breadth, salience, and the standardizability of addiction-related cues. However, the use of VR environments in addiction research is still at its early stages, but with a very promising future.

VR technologies facilitate the investigation of environmental triggers [12]. They enable to differentially assess the effects of cues on behavior and biology of individuals with addictive behaviors. Cues are divided into multisensorial proximal cues, which refer to specific objects directly related to drug use or addictive behavior (e.g., sight and smell of a preferred alcoholic beverage, a cigarette, syringes), contextual environmental cues (e.g., convenience store) with or without social interaction, and complex cues, i.e., how proximal and contextual cues interact with each other to influence addictive behavior [12–15]. VR technology provides an excellent basis to investigate the effects of various combinations of proximal and contextual cues, which is important because preclinical evidence suggests that contexts may influence drug-seeking behavior independently from the direct cues [16]. Cue exposures in VR settings are more potent and reach higher ecological validity than traditional methods to evoke craving [17]. In particular, VR simulations applied via head-mounted displays (HMDs), goggles, or a CAVE, i.e., projection wall systems, are promising tools [18].

VR simulations are already successfully employed in exposure-based treatments of other mental illnesses such as anxiety disorders [19] and posttraumatic stress disorder [20]. A recent review of literature from the addiction field has shown that VR simulations can elicit craving, although treatment effects have been found to be heterogeneous [21].

In this article, our aim is to provide a review on the available studies using VR technology via HMDs, goggles, or a CAVE system to enlighten the mechanistic underpinnings of addictive behaviors. Our systematic literature search identified 38 studies. We discuss the advantages of VR simulations over traditional laboratory and clinical settings, the challenges and potentialities linked to the use of VR, and the shortcomings of the available literature. We propose future directions of VR research on addictive behaviors.

## Methods

**Fig. 1:**
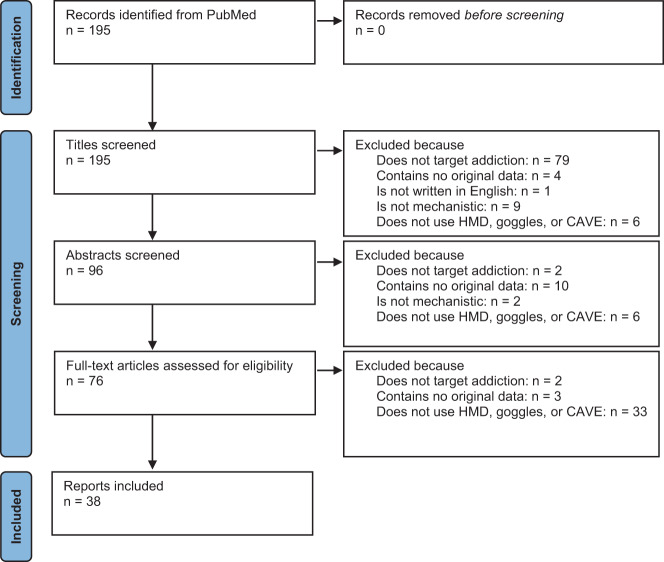
PRISMA flow diagram of the study selection process. It illustrates the numbers of records identified, excluded and included, and reasons for exclusions. HMD head-mounted display.

The literature search was conducted on March 17, 2021, and the following search term was used in PubMed (https://pubmed.ncbi.nlm.nih.gov/: (virtual[Title]) AND ((addictive[Title]) OR (addiction[Title]) OR (substance[Title]) OR (alcohol[Title]) OR (cocaine[Title]) OR (cannabis[Title]) OR (opioid[Title]) OR (tobacco[Title]) OR (nicotine[Title]) OR (methamphetamine[Title]) OR (gaming[Title]) OR (gambling[Title])). The eligibility criteria were addiction as a relevant target, original data, published in English language, clarification of mechanisms underlying addiction, and use of HMD, goggles, or CAVE. We excluded studies without evidence for use of HMD, goggles, or a CAVE setting because these immersive technologies provide higher ecological validity with stronger effects. The studies were screened and selected independently by two researchers (MM and BL), differences were discussed, and in all cases a compromise was agreed on. Out of the initially identified 195 records, 38 studies could be included (see Fig. 1 for the PRISMA diagram and Table 1 for the selected studies) and the reported results were divided into the following categories: craving, psychophysiology and affective states, attention and cognition, brain activity, other treatment mechanisms, and study protocols. Table 1 shows also the country where the study was conducted, the sex composition, average age, and whether a social component has been included in the experiment. This systematic review was not pre-registered.

**Table 1:** VR studies according to treatment mechanism addressed.

Publication		Target behavior	Mechanism	Country	Case number	Mean age (years) ± SD	Social component	VR Technique
Lee et al. (2003)	[34]	Cigarette smoking	C	Korea	22 (0% ♀)	Not reported	+	HMD
Bordnick et al. (2004)	[35]	Nicotine dependence	C	USA	13 (69% ♀)	37.1 ± 12.2	+	HMD
Lee et al. (2004)	[41]	Cigarette smoking	C, PA	Korea	16 (0% ♀)	17.1 ± 0.8	+	HMD
Saladin et al. (2006)	[46]	Crack cocaine dependence	C, PA	USA	11 (55% ♀)	42.1 ± 7.3	+	HMD
Carter et al. (2008)	[37]	Nicotine dependence	C	USA	22 (% ♀ not reported)	20.8 ± 1.4	+	HMD
Bordnick et al. (2008)	[26]	Alcohol use disorder	C, CBA	USA	32 (20% ♀)	39.5 ± 10.1	+	HMD
Lee et al. (2009)	[30]	Alcohol dependence	CBA	Korea	20 VR (0% ♀) 18 CBT (0% ♀) 15 controls (0% ♀)	38.6 ± 5.9 37.5 ± 4.6 38.6 ± 4.6	+	Goggles
Moon and Lee (2009)	[43]	Cigarette smoking	CBA	Korea	8 (0% ♀)	17.0 ± 0.8	+	HMD
Bordnick et al. (2009)	[47]	Cannabis abuse or dependence	C, CBA	USA	20 (20% ♀)	26.8 ± 6.7	+	HMD
Girard et al. (2009)	[64]	Cigarette smoking	O	Canada	91 (57% ♀)	44 ± 11	—	HMD
Lee et al. (2008)	[29]	Alcohol dependence	C	Korea	14 patients (0% ♀) 14 controls (0% ♀)	39.6 ± 6.0 36.8 ± 7.4	+	HMD
Ferrer-García et al. (2010)	[38]	Cigarette smoking	C	Spain	27 (31% ♀)	29.7 ± 13.4	+	HMD
Traylor et al. (2011)	[12]	Nicotine / alcohol dependence	C	USA	14 double dependents (29% ♀) 7 nicotine dependents (57% ♀)	38.4 ± 9.7 39.3 ± 11.0	+	HMD
Choi et al. (2011)	[33]	Nicotine dependence	C, PA	Korea	10 (10% ♀)	26.2 ± 5.1	+	CAVE
Acker and MacKillop (2013)	[36]	Nicotine dependence	C	USA	47 (39% ♀)	28 ± 10.8	—	HMD
Giroux et al. (2013)	[51]	Gambling	C	Canada	10 (40% ♀)	63.4 ± 7.2	+	HMD
Park et al. (2014)	[42]	Nicotine dependence	C	Korea	15 CET (0% ♀) 15 CBT (0% ♀)	30.7 ± 6.6 33.1 ± 5.5	+	CAVE
Kaganoff et al. (2012)	[40]	Nicotine dependence	C, CBA	USA	46 (48% ♀)	46.9 ± 9.3	+	HMD
Park et al. (2015)	[53]	Gambling	C, PA	Korea	12 (0% ♀)	32.3 ± 6.4	+	CAVE
Thompson-Lake et al. (2015)	[25]	Nicotine dependence	C, PA	USA	36 (39% ♀)	Not reported	+	HMD
Bordnick et al. (2012)	[39]	Nicotine dependence	C, CBA	USA	25 replacement therapy (40% ♀) 21 VR skill training (57% ♀)	46.2 ± 8.4 47.9 ± 10.4	+	HMD
Park et al. (2016)	[63]	Excessive internet gaming	CBA	Korea	12 gaming addiction (VRT, 0% ♀) 12 gaming addiction (CBT, 0% ♀) 12 casual gaming (0% ♀)	23.6 ± 2.7 24.2 ± 3.2 23.3 ± 2.9	+	Goggles
Giovancarli et al. (2016)	[65]	Nicotine dependence	SP	France	Not reported	—	+	HMD
Bouchard et al. (2017)	[52]	Gambling	C, CBA	Canada	28 frequent and 36 infrequent (% ♀ not reported) 34 pathological (35% ♀) 25 pathological (50% ♀)	Not reported 45 ± 12.6 47 ± 12.8	+	HMD HMD HMD
Shin et al. (2018)	[50]	Internet gaming disorder	C	Korea	34 Internet gaming disorder (0% ♀) 30 controls (0% ♀)	17.2 ± 4.6 18.6 ± 5.0	+	HMD
Chen et al. (2018)	[67]	Stimulant addiction disorder	SP	China	180 (% ♀ not reported)	—	—	HMD
Wang et al. (2018)	[48]	Methamphetamine dependence	C, PA	China	61 abstinent (0% ♀) 45 controls (0% ♀)	33.6 ± 7.3 34.3 ± 8.6	+	HMD
Wang et al. (2019)	[49]	Methamphetamine dependence	C, PA	China	31 intervention (0% ♀) 29 waiting list (0% ♀) 612 intervention (17% ♀) 276 waiting list (25% ♀)	35.0 ± 7.5 32.6 ± 6.6 34.0 ± 7.6 33.4 ± 7.8	+ +	HMD HMD
Ghiţă et al. (2019)	[27]	Alcohol use disorder	C	Spain	13 AUD (38% ♀) 14 social drinkers (86% ♀)	48 ± 4.8 23 ± 5.6	+	HMD
Tan et al. (2019)	[44]	Methamphetamine use disorder	C, PA, CBA	China	60 (0% ♀)	35.4 ± 5.6	+	HMD
Simon et al. (2020)	[28]	Heavy alcohol drinking	C	Belgium	18 heavy drinkers (44% ♀) 21 occasional drinkers (52% ♀)	24.6 ± 3.1 23.7 ± 2.9	+	HMD
Liu et al. (2020)	[66]	Methamphetamine use disorder	SP	China	95 (% ♀ not reported)	—	Not reported	HMD
Ding et al. (2020)	[58]	Methamphetamine dependence	PA, CBA	China	333 dependents (0% ♀) 332 controls (0% ♀)	33.8 ± 6.5 33.6 ± 7.9	+	HMD
Hernández-Serrano et al. (2020)	[31]	Alcohol use disorder	C	Spain	15 VR-CET + TAU and 27 TAU (50% ♀)	54.6 ± 7.7	+	HMD
Amato et al. (2020)	[61]	Alcohol effects	CBA	Australia	54 (56% ♀)	20.7 ± 2.1	+	HMD
Lee et al. (2020)	[59]	Internet gaming disorder	PA	Korea	23 addicted (0% ♀) 29 controls (0% ♀)	18.9 ± 3.9 19.8 ± 3.9	+	HMD
Tsai et al. (2021)	[45]	Methamphetamine use disorder	C, PA	Taiwan	26 addicted (19% ♀) 11 controls (36% ♀)	Not reported	+	HMD
Ghiţă et al. (2021)	[32]	Alcohol use disorder	PA, CBA	Spain	1 addicted (0% ♀)	49	+	HMD

Social component refers to social interaction or pressure, animate video, or presence of an avatar.

*C* craving, *CBA* cognition and brain activity, *CBT* cognitive behavioral therapy, *CET* cue-exposure therapy, *HMD* head-mounted display, *O* others, *PA* physiology and affective states, *SP* study protocol, *TAU* treatment as usual, *VR* virtual reality, *VRT* VR therapy.

## Results

### Craving

A plethora of complex pathogenetic models including affective and cognitive processing, conditioning learning, motivational processes, and the incentive sensitization theory have been associated with craving [22, 23]. Environmental cues trigger craving, which increases the risk for relapse [24]. However, the complexity of this model should not be underestimated, as craving is elicited by an interaction of various individual and environmental factors including affective states, autobiographical information, and sensory input. VR simulations offer dynamic and complex exposure settings, which is a prerequisite to gain a more in-depth understanding of which factors trigger craving [25]. This might result in novel treatment strategies. For example, cue reactivity in VR simulations might reliably predict who will relapse in the future or who will respond to cue exposure therapy.

#### Alcohol

VR simulations provoke alcohol craving in subjects with alcohol abuse or dependence [26]. Diverse VR environments such as at home, bar, restaurant, and pub elicit alcohol craving in patients with alcohol use disorder (AUD), but not significantly in social drinkers [27]. In VR, heavy alcohol drinkers exhibit higher craving scores than occasional drinkers and the extent of ecological validity correlates with craving scores in heavy, but not in occasional drinkers [28]. In patients with alcohol dependence, Lee et al. [29] investigated the interaction between cue-laden and cue-free environments, social pressure, and group affiliation “addicted patients vs. controls” on induction of craving. In the cue-free VR environment, social pressure, i.e., persuasions to consume alcohol, increased craving in both patients and controls, whereas in the cue-laden VR environment, social pressure enhanced craving in the controls but not in the addicted group, as the latter experienced already high levels of craving due to the environmental cues. In a VR project on cross-cue reactivity, nicotine-dependent and non-alcohol-dependent subjects showed lower alcohol craving than nicotine- and alcohol-dependent subjects in an office environment, but not in a party simulation [12]. This observation suggests that non-alcohol-dependent smokers are more vulnerable to contextual cues. With regards to treatment effects, ten sessions of VR treatment reduced craving of alcohol-dependent patients more strongly than treatment as usual consisting of education and cognitive therapy [30]. In a single-blinded randomized trial, treatment as usual combined with six VR cue exposure therapy sessions over a period of approximately five weeks was superior to treatment as usual alone in reducing craving from admission to discharge in patients with AUD [31]. Notably, the study results also indicate that VR cue exposure therapy might be more efficacious in patients with higher than in those exhibiting lower levels of craving. The effectiveness of VR cue-exposure therapy has also been shown in a case report [32]. Taken together, alcohol craving is elicited in cue-laden VR environments; the effects differ between patients with AUD and social drinkers, depending on the social context, and are affected by cross-cue reactivity. Moreover, preliminary evidence suggests that VR cue exposure therapy might excel traditional methods.

#### Tobacco

Similar to alcohol, VR simulations reliably induce craving for smoking [33]. Smoking cues in VR environments increase craving for cigarette smoking more strongly than cues presented in pictures [34] or than non-smoking or neutral cues [12, 25, 35–37]. Craving for smoking is induced by various environments including at home, pub, restaurant, and street scenarios [38]. Interestingly, the subjective level of presence correlates positively with craving for smoking a cigarette [38]. Accordingly, craving is induced less strongly in a paraphernalia room containing sights, sounds, and scents linked to tobacco smoking than in a party room with similar smoking cues plus interacting avatars that offer cigarettes, drinks being poured, conversations, and cigarettes being lit up [25]. Craving for cigarette smoking was found to be more persistent and to show a carry-over effect in the neutral condition in alcohol-dependent smokers, but not in non-alcohol-dependent smokers [12]. In treatment studies, VR cognitive behavioral therapy for cigarette smokers reduces craving [39, 40]. Six sessions of cue exposure therapy also reduced the morning smoking count [41]. Moreover, VR cue-exposure therapy and cognitive behavioral therapy are effective for smoking cessation, although the intervention effects on craving only showed a trend in one study [42]. However, also null findings of cue-exposure therapy on craving have been reported [43]. Altogether, VR simulations with smoking cues induce craving, and, similar to the alcohol field, repeated VR cue exposure reduces craving for smoking.

#### Illicit drugs

Also in users of illicit drugs, drug-related cues elicit craving [44, 45]. VR simulations which depict the use of cocaine and which allow for interacting with a dealer increase craving in cocaine-dependent individuals [46]. VR marijuana environments elicit craving in individuals with cannabis abuse or dependence with even large effect sizes [47]. In patients with methamphetamine dependence, cue-induced craving is associated with cue-elicited heart rate variability changes [48], and a counterconditioning approach to pair methamphetamine-related cues with aversive stimuli (e.g., contact to the police, hallucinations, infections, death) reduces craving and liking of methamphetamine (in comparison to a waiting list) [49].

#### Internet gaming and gambling

In a VR Internet café simulation, patients with internet gaming disorder (IGD) develop higher craving than controls. Interestingly, the simulations of entering an internet café and being invited to game result in higher craving than observing a conversation about internet games. Moreover, craving was lower during a cognitive-behavioral-oriented refusal skill practice than during the invitation to game condition [50]. In gamblers, being on the sidewalk of a street and seeing a bar elicit craving, which highlights the importance of contextual stimuli [51]. The VR simulation also provokes craving more strongly in frequent than in infrequent gamblers with effect sizes comparable to a real video lottery terminal [52]. Different VR casino environments increase craving also in recreational gamblers [53]. The effect was strongest in the scenario of playing a casino game, and the authors speculate that higher presence relative to the other scenarios (navigating the casino, choosing chips exchanged, witnessing a jackpot scene and discussion about gambling) might account for this observation. However, a single 20-minutes exposure was not long enough to induce extinction [51]. These data show that VR simulations can induce craving also in individuals with non-substance-related addictions. Moreover, they highlight the diverse behavioral effects of complex cues and underline the need for experimental research using VR technologies.

### Physiology and affective states

Perceived stress, depressed mood, and anxiety are important factors in the development and maintenance of SUD [54, 55], and anxiety is involved in eliciting craving [56]. VR studies used skin conductance rate, which might serve as an objective mirror of affective states [57], and heart rate variability to investigate the role of the autonomic nervous system and emotions in addiction.

#### Alcohol

Cue exposure induces anxiety in patients with AUD, whereas it decreases anxiety in social drinkers, and these cue-elicited anxiety effects differentiate better between patients and social drinkers than cue-elicited craving levels [27]. In a case report of a male patient with long lasting severe AUD, six cue-exposure sessions reduced cue-elicited anxiety levels by 95% [32].

#### Tobacco

In nicotine-dependent subjects, heart rate is increased by smoking cues (relative to neutral cues) including an avatar lighting up a cigarette or offering a cigarette to the participant and a burning cigarette in an ashtray, but not viewing a cigarette pack on the table [25]. This indicated that animated simulations excel unanimated simulations. Smoking cues also heighten skin conductance levels and social situations increase skin conductance levels more than object cues related to cigarette smoking [33]. Moreover, the psychophysiological response in skin conductance decreases with repeated exposures [33].

#### Illicit drugs

Cocaine-related VR simulations increase heart rate and reduce happiness in cocaine-dependent individuals [46]. In male patients with methamphetamine use disorder (MUD), VR methamphetamine-cue exposure induces higher skin conductance than the neutral condition [44]. Furthermore, VR cue exposure increases skin conductance more strongly in male patients with MUD than in healthy controls. In this project, galvanic skin response was also superior to electroencephalogram (EEG) signals to classify patients vs. controls [58]. In men with methamphetamine dependence, the methamphetamine-cue VR exposure induces larger heart rate variability in affected subjects, while it reduces the heart rate variability in healthy controls [48]. Also in a Taiwanese study, heart rate variability, galvanic skin response, and pupil size changed from pre to post VR stimulation in patients with MUD, but not or only weakly in controls. Patients differed significantly from controls post VR simulation in heart rate variability and skin conductance [45]. These data agree with emotional arousal during VR simulation in patients with SUD, but not in controls. Concerning the potential therapeutic outlook, a counterconditioning procedure in a VR environment showed larger decreases in heart rate variability indexes on time domain and non-linear domain pre to post intervention than a waiting list group [49].

#### Internet gaming and gambling

In men with IGD, stronger anxiety and depression correlate with more severe IGD and a higher percentage of digital activities [59]. However, electromyography, skin conductance, and heart rate did not significantly change when recreational gamblers were exposed to VR casino scenarios [53]. The authors speculate that the missing effect was due to the specific study population, not fulfilling the dependence criteria, but reporting recreational use.

#### Summary

Taken together, addiction-related cues in VR environments increase skin conductance in subjects with addictions (relative to neutral conditions and relative to controls). Because the skin conductance mirrors autonomous nervous system activity, these results highlight a more stressful state under VR cue exposure. Moreover, addiction-related cues in VR environments also affect heart rate, which can give insight into regulation of emotional load [60], and anxiety and depression levels. Finally, animated simulations and the inclusion of social situations may reinforce the effects on physiology and affective states, probably due to higher immersion and presence.

### Attention and cognition

VR simulations with embedded neuropsychological assessments can reliably and sensitively measure attention, decision-making, and visual processing speed [61]. In patients with AUD, addiction-related cues in VR attract more attention to the sight, the smell, and the thoughts about alcohol than neutral cues [26]. In a male patient with severe AUD, six VR cue exposure sessions reduced interference towards alcohol content in the Alcohol Stroop Task [32]. A treatment study applying cognitive behavioral therapy in a VR setting to tobacco smokers showed higher attention to visual and olfactory cigarette cues and increased thoughts about smoking in cue-enriched conditions than in a neutral condition [40]. Moreover, the intervention increased smoking abstinence self-efficacy and confidence to resist smoking and reduced the number of cigarettes smoked during and until two months post treatment [39]. In marijuana VR environments compared to neutral environments, individuals with cannabis abuse or dependence are more likely to pay attention to the sight, the smell, and thoughts of cannabis [47]. A study in pathological gamblers highlights the feasibility to use VR in cognitive behavioral therapy. In comparison to imaginal exercises, the VR setting facilitates the identification of risk situations for gambling and tends to detect more dysfunctional thoughts [52]. Altogether, the identified studies show that VR settings are suitable to investigate how substance use affects cognitive processes and how addiction-related cues attract attention and thinking. Therefore, VR may serve as an important tool to investigate cognitive dysfunctions in patients with addictions.

### Brain activity

It is well established that dysfunctional brain activities play an important role in addictive behaviors. VR technologies have been combined with EEG and functional magnetic resonance imaging (fMRI) to provide insight into treatment mechanisms of addiction.

#### Alcohol

In alcohol-dependent patients, ten sessions of VR therapy increased absolute EEG alpha power in Fp2-A2 and F8-A2. No such effects were observed after treatment as usual, which consisted of education and cognitive behavioral therapy elements [30].

#### Tobacco

In tobacco smoking men, smoking cues display stronger fMRI brain activity than neutral cues in the prefrontal cortex (superior frontal gyrus, right medialfrontal gyrus, left orbital gyri), left anterior cingulate gyrus, right superior temporal gyrus, left uncus, right fusiform gyrus, right lingual gyrus, and right precuneus. The authors also report a reduction of the activity in the left superior and inferior frontal gyrus pre vs. post VR cue-exposure therapy [43].

#### Illicit drugs

In patients with MUD, low and high gamma EEG bands in the right dorsolateral prefrontal cortex are weaker in a methamphetamine-cue VR condition compared to a neutral condition, and the EEG bands predict skin conductance levels [44]. The alterations in dorsolateral prefrontal cortex function are related to reduced impulse control ability [62], which is of direct relevance to addictive behavior.

#### Internet gaming

An 8-week VR therapy consisting of relaxation, simulation of high-risk situations, and sound-assisted cognitive reconstruction reduced the severity of online gaming addiction in young men, and the effect size was similar to the cognitive behavioral therapy condition. VR therapy enhanced the functional connectivity in the left middle frontal–posterior cingulate cortex–bilateral temporal lobe and resulted in improved balance in the cortico-striatal–limbic circuit. This suggests that VR therapy prevents habitual, emotionless gaming in that it facilitates limbic-regulated responses to rewarding stimuli [63].

#### Summary

Taken together, VR studies using EEG and fMRI technology show impaired activity of the prefrontal cortex in patients with addictive disorders in comparison to control subjects; also, these alterations are linked to psychophysiological parameters and change during VR therapy.

### Other treatment mechanisms

Girard et al. [64] compared the interventions “crushing cigarettes” vs. “grasping balls” in a VR environment (12-week study period). Crushing cigarettes was superior to reduce Fagerström scores for severity of nicotine dependence, decrease number of cigarettes smoked, and reach abstinence. In terms of behavioral economics, VR tobacco cues relative to neutral cues have been shown to increase willingness to invest more money in cigarettes, to continue smoking in case of high prices for cigarettes, and to decrease sensitivity to cigarette prices [36].

### Study protocols

The systematic literature search also identified three study protocols to investigate the effects (i) of cognitive behavioral therapy and VR exposure therapy on abstinence rates, anxiety, and depression in tobacco smokers [65], (ii) of a VR-based memory retrieval-extinction intervention on craving, psychophysiology, affective states, and brain activity in men and women with MUD [66], and (iii) of mindfulness and VR cue exposure on craving, anxiety, depression, and attention bias in MUD [67].

## Discussion

To our knowledge, this is the first review based on a systematic literature search of studies using VR HMDs, goggles, or CAVE technology to provide insight into treatment mechanisms underlying addictive disorders. The available body of research shows that VR environments are a reliable technology to provoke craving and physiological reactions and to influence affective states, attention, cognition, and brain activity. These effects have been shown for both substance-related and for non-substance-related addictive behaviors. Our predefined search criteria lead to the exclusion of studies on conditions such as eating disorders and morbid obesity. However, the existing literature suggests that the above reported effects are also present in food addiction [68–71] highlighting the generalizability to a broad range of addiction-related behaviors.

Furthermore, VR environments have the potential to excel traditional laboratory and clinical methods regarding effect size, as more immersive simulations with a higher sense of presence are more powerful. The effects also depend on the characteristics of the study populations (i.e., addicted individuals, heavy users, social users), the social context, as well as the integration of social interaction into the VR simulation. There is also preliminary evidence for cross-cue reactivity. Finally, repeated exposures appear to reduce psychophysiological reactivity. Various therapeutic strategies including VR cue exposures, a VR counterconditioning approach, and VR cognitive behavioral therapy addressing self-confidence and coping skills have been tested with mixed results. Study protocols describe ongoing projects using VR exposure therapy [65], VR-based memory retrieval-extinction intervention [66], and mindfulness [67]. However, further experimental research using VR technologies is necessary.

### Advantages of VR simulations over traditional laboratory, clinical, and real-life settings

Cue exposure in VR simulations excels cue exposure in traditional laboratory or clinical settings in several ways including higher effect size, easier and more realistic bench-to-bedside translation, higher level of standardization, easier repeatability, reduced distraction, higher immersion and presence, and higher feasibility of studies on complex cues and complex target behaviors. VR environments elicit stronger effects than traditional laboratory or clinical settings based on pictures [17, 34]. fMRI data gathered during visually three-dimensional conditions also evoke more attention and visual balance than data from visually two-dimensional conditions [72]. Moreover, VR environments facilitate the translation from experimental research to patient care as they offer highly standardized and repeatable procedures without variation between experimental research settings and patient care settings. This might be particularly relevant to stress paradigms, which play an important role in addictive disorders. Once the VR environments are established, there are only low costs and the training setups are widely accessible, even from the home setting. However, there is still great need for research on how exactly the novel VR environments can be effectively translated to the home environment.

VR environments can simulate reality without limitations to space and time. VR also permits to create environments that would be unethical, illegal, or dangerous in real-life. While environmental cues of interest can be specifically inserted, unexpected and unwanted distracting effects, such as surrounding light and sounds could be filtered out easily.

Patients are more willing to accept exposure therapy and reduce avoidance behavior when conducted in VR instead of a real-life setting, as VR exposure is estimated less aversive. Especially to individuals with SUD, VR technology seems very attractive [46]. Addictive disorders are highly co-morbid with attention-deficit hyperactivity disorder [73] and social phobia [74]. Subjects with attention deficits and/or discomfort in groups might particularly benefit from VR therapies, as these might immerse such patients more easily in the therapy as compared to traditional cognitive behavioral therapy elements without VR simulations [75].

VR simulations facilitate the combination of different proximal cues with contextual cues, thus providing an excellent environment to study reactivity to complex cues. This does not only include visual and auditory cues, but also refers to actions and social factors, such as facial expressions and social interaction [48]. The investigation of how proximal, contextual, and social factors interact with each other is important, since for example in smokers greater craving is elicited by visual and olfactory cues [76], contextual cues [77], and interpersonal interaction [78]. Moreover, standardization of social interaction is a challenge in laboratory experiments. However, social situations related to substance use have been shown to elicit even stronger psychophysiological reactions than object exposure alone [33]. Lee et al. [29] have highlighted the relevance of studies exploring the interactions between different cues and social interactions. They established a ceiling effect in that social pressure affects craving for alcohol in controls independent from whether environmental alcohol-related cues are present or not, whereas in addicted subjects, social pressure increases craving for alcohol in a cue-free environment, but not in a cue-laden environment. Thus, VR technology is an excellent tool to significantly advance our understanding of how social interactions contribute to the development and maintenance of addictive behaviors.

In traditional laboratory studies, addiction phenotypes are often assessed outside of the settings following the exposures, e.g., stress paradigms. In contrast, VR experiments enable to embed recording of behavioral, psychophysiological endpoints directly in the VR display system. This allows for real-time event-triggered data analysis. An example of an embedded VR tool is the CONVIRT test battery, which allows to assess the neuropsychological functions of attention, decision-making, and visual processing speed within the VR simulation [61]. This tool also enables to measure saccadic eye movements.

Furthermore, in comparison to traditional laboratory studies to assess drug craving, effects, and self-administration [79, 80], VR experiments allow researchers to modify environmental factors, such as the bar appearance, more easily. In addition, addictive substances are not only consumed in bars, but also at home (e.g., living room setting), as well as in public locations (e.g., open air settings), or with friends in a wide array of private locations or clubs. All these settings can be easily simulated using VR, which grants a wider study of cue-elicited reactivity in more settings than just the bar laboratory paradigm. However, a limitation is that as of yet we still lack empirical evidence for the differences in effect size between VR settings and bar laboratory exposures. In the future, more direct comparisons are needed to figure out differences in effects between VR environments and traditional laboratory settings, clinical settings, and real-life settings.

### Challenges of using VR technology

Although VR technology excels with high ecological validity, the effect sizes of VR simulations depend on their capability to immerse subjects and to create high levels of presence [81]. Thus, perceived ecological validity is a relevant factor when studying cue reactivity in VR simulations. For example, perceived realism predicted craving post VR stimulation in heavy alcohol drinkers [28]. The perception of presence also depends on functional fidelity [18]; i.e., the ability of users to interact realistically with the environment. Presence has also been highlighted as an important factor for intervention efficacy [64]. In a study on IGD, conditions where participants had to follow a given pathway (internet café entrance task) and where they interacted with avatars (gaming invitation task) evoked stronger craving than a passive condition (observing a conversation about internet games) [50]. Accordingly, a higher subjective level of presence in VR relates to more craving for smoking a cigarette [38]. These data encourage researchers to use VR environments that address multiple sensory systems, include social interaction, and request active participation. The implementation of verbal and non-verbal reactions of avatars to the participants’ behaviors and a complex interactivity are limited due to technical constraints. Programming such interactive simulations might be very time consuming and requires expert skills. However, it is an important goal to create VR simulations with avatars that provide appropriate reactions. The establishment of multidimensional VR settings addressing different sensory systems to create the highest realism and naturalism possible has only just begun. The literature stimulates to believe that future efforts addressing this issue will generate important progress.

Another problem of VR simulations might be “cybersickness”, which refers to nausea, headaches, vomiting, and spatial disorientation and stems from differing visual, vestibular, and proprioceptive information. Such symptoms might limit the ecological validity. However, a study found that VR was not significantly associated with relevant cybersickness [52], and the use of HMDs is more likely to cause cybersickness than VR presented in a CAVE [33]. Also, dropout rates in the VR setting have been only slightly higher than in the non-VR control environments [39], which indicates that VR settings pose only a low additional burden on the participants. Taken together, there is so far no compelling evidence that cybersickness or safety concerns might pose a significant problem to strategies based on VR technology. Nevertheless, future studies are needed to investigate feasibility, acceptability, and safety of VR simulations in the light of adverse events. This is particularly important, as the potential of translation into clinical settings strongly depends on the ability of patients and their clinicians to use novel tools easily, effectively, and without harm.

To establish classification procedures, advanced algorithms of artificial intelligence and machine learning techniques are needed to analyze the multidimensional and longitudinal data gathered in VR experiments [82]. Previous attempts have been successful. Using a split-dataset design, Ding et al. [58] trained several algorithms on EEG and skin conductance data to distinguish male patients with MUD from male controls and found good classification accuracies for a validation dataset (88.57 for random forest, 90.38 for support vector machine, and 90.68 for logistic regression classification).

### Potential of VR technology for treatment

It is a well-known problem that psychotherapeutic treatments have only limited efficacy when applied in a clinical setting or a therapy practice. Therapeutic interventions might be more effective in VR environments as these offer more realistic high-risk situations as compared to the relatively artificial clinic settings (that often do not allow to bring the patients into high-risk environments such as bars) [39]. Therefore, VR interventions are likely to increase the effectiveness of conventional cue exposure therapies [21]. VR settings are also effective as a tool to address factors of cognitive behavioral therapy such as self-confidence and coping skills. For example, refusal skills practice in a VR internet café environment reduces craving in patients with IGD [50]. VR may create realistic environments for treatment, which could not be used in natura due to safety or confidentiality concerns.

In clinical settings, it is often difficult to identify the exact individual triggers. Dysfunctional behaviors can sometimes only be provoked with reduced severity. Moreover, health care professionals often have difficulty helping the patient to transfer newly learned skills to the natural setting. VR technologies have the potential to overcome these hurdles as they offer access to real-world environments that may make cue exposure trainings more effective. Hence, VR technology allows the therapist to provide advice in a natural-like setting. Therapists found also other advantages in integrating VR into cognitive behavioral therapy, such as easier access to triggers, emotions, and dysfunctional thoughts particularly in highly rational patients, more efficient validation of what has been learned during therapy, and stronger reinforcement of self-efficacy [52]. Moreover, specific settings can be repeated without any variance between the training sessions to test (and retest) the newly developed behaviors. Contrary to the traditional settings, VR simulations also allow for easy switches between scenario difficulties. Due to the computer-controlled presentation of stimuli and timing, VR technology enables higher standardization than human-operated experimental paradigms. VR cue exposure-induced changes of cue-reactivity (craving and anxiety) also generalize into daily life experiences, which is a very important goal when aiming at tailoring effective treatment strategies [32].

VR settings enable precisely defined exposures to stereoscopic three-dimensional and interactive stimuli with high ecological validity under strictly controlled conditions. The therapeutic sessions can be repeated in a standardized manner as often as feasible, which is an important factor for cue-exposure training sessions in a confidential therapeutic setting. The VR settings can also be easily adapted to individual needs such as combinations of drug-related cues and multi-sensorial contextual cues. For example, custom-made VR environments may allow the therapists and patients to select individual types of alcoholic beverages and drinking situations during the sessions. This aspect might also be particularly relevant to the generalization of exposure effects and reduction of renewal risk in the context dependency of extinction [83]. VR simulations might also contribute to personalized medicine, as they enable to easily provide additional input or adapt the cues to the needs of the individual (e.g., embodiment projects). VR training sessions that include interactive behavior may be used to reveal and modify automatized addictive behavior [64].

The costs to build up and operate a VR system are a major issue. However, after establishment, treatments based on VR simulations are simple to use and cause only low costs. Thus, such therapeutic methods can be easily implemented in daily clinical routine. In the last decade, software and hardware have been well advanced, and hardware companies now intend to increase production for the mass market. The costs have been decreasing, and the availability of HMDs will increase in the near future. As a result, these methods may be used to practice psychotherapeutic methods at home.

### Limitations

The available literature is limited in several ways. So far, the investigated sample sizes are mostly small. Future research is needed to study different contexts, sexes, ethnicities, and age groups to test for generalizability. Most of the studies focus on men; women are strongly neglected, which is a problem of addiction research in general [84]. Also, they rather include young cohorts, as digital natives are more enthusiastic to use modern digital technology. However, treatments in VR might be especially interesting to older people who are less mobile. Further open questions are which of the effects found in VR studies can be generalized and which are restricted to subgroups of substance-related and non-substance-related addictive behaviors. Moreover, the available studies have mostly focused on self-reported outcomes such as craving and affect. It would be important for future research to integrate additional objective measures such as physiological parameters and neuro-imaging phenotypes more regularly. Studies using VR in addiction research have mostly addressed VR environments with pleasant connotations (such as enjoying drug use together with the peer group); however, stress situations are also well-known reasons for substance use and can trigger addictive behaviors. Thus, future studies should also investigate negative situations, e.g., stress paradigms.

The first VR technologies were developed in the 1960s, and since the 1980s, some of the VR systems were already highly sophisticated, with full stereo display, head tracking, and at least hand tracking [85]. However, since then VR technologies have made strong progress in aspects such as graphics quality, computing power, and accessibility. As higher levels of presence produced by VR environments with higher capabilities for immersion are thought to produce stronger effect size [81], direct comparisons of early and more recent VR studies need to be interpreted carefully due to potential biases induced by the technological progress.

## Outlook

### The Proteus effect: embodiment

The “Proteus effect” refers to an interesting phenomenon characterized by one’s change in behavior and cognitions depending on the avatar that one embodies. Embodiment can be achieved by visually substituting one’s own body with a virtual one through visuomotor synchrony (the real-time motion capture applied to the virtual body) as well as through visuotactile stimulation, in which there is a correspondence between a virtual object seen to touch the body and a synchronous touch on the real body [86]. For instance, white participants who embody dark-skinned virtual bodies show a reduction in implicit bias against black people [87]. Also, embodiment techniques are able to change temperature sensitivity [88]. Finally, embodying self-compassion within VR may have a considerable clinical potential in the treatment of depression and may decrease SUD risk [89]. Thus, avatars inducing a sense of self-confidence might promote desired therapeutic effects.

### Eye-tracking

With the advent of eye-tracking technology incorporated into VR HMDs, a new avenue of research on addicted patients’ attention gaze shifts is born. Indeed, studies on attentional bias in addiction have tasks that are incongruent with the natural environment [28]. This problem might be overcome by novel eye-tracking technology. Moreover, specific background cues that induce craving can be assessed by tracking eye movements in virtual supermarkets, which have already been employed for marketing purposes [90]. Recently, Tsai et al. [45] developed a VR system for the purpose of inducing cravings for MUD patients and observed significant differences in eye tracking between pre-VR stimulation and post-VR stimulation in MUD patients but not in controls.

### Neuropeptides, genetics, and epigenetics

Whether VR immersion impacts on neurobiological parameters involved in the pathogenesis of addictive behaviors, such as cortisol, oxytocin and vasopressin, is yet to be determined. Moreover, research on how genetics and epigenetics interact with behaviors in VR is needed. To our knowledge, only few studies applying VR technology have observed changes in neuroendocrine parameters [91, 92]. To investigate whether such neuroendocrine effects in VR can be observed in patients with addictions can help us elucidate key neurobiological underpinnings of the etiopathogenesis of addiction.

## Conclusion

VR is a powerful and promising tool for the study of cue-elicited craving in patients with addictions. It is a technology characterized by an exponential growth with important implications for the development of addiction research. VR can be associated with other technologies, such as eye-tracking technology, in order to pin-point specific cues that elicit craving in patients. It may also help us differentiate cue-elicited cravings among different types of addictions. Finally, changes in neuroendocrine parameters elicited by VR environments may aid in further elucidating the neurobiological underpinnings of addiction. VR is already complementing naturalistic settings in addiction research, increasing the feasibility and reproducibility of studies of cue-elicited cravings. Future VR studies will also contribute significantly to the development of new therapeutic approaches.
